# A chemosynthetic weed: the tubeworm *Sclerolinum contortum* is a bipolar, cosmopolitan species

**DOI:** 10.1186/s12862-015-0559-y

**Published:** 2015-12-14

**Authors:** Magdalena N. Georgieva, Helena Wiklund, James B. Bell, Mari H. Eilertsen, Rachel A. Mills, Crispin T. S. Little, Adrian G. Glover

**Affiliations:** Life Sciences Department, Natural History Museum, London, UK; School of Earth and Environment, University of Leeds, Leeds, UK; School of Geography, University of Leeds, Leeds, UK; Centre for Geobiology, University of Bergen, Bergen, Norway; Department of Biology, University of Bergen, Bergen, Norway; Ocean and Earth Science, National Oceanography Centre Southampton, University of Southampton, Southampton, UK

**Keywords:** Siboglinidae, Polychaeta, Annelida, Antarctica, Gene flow, Deep-sea, Connectivity, Hydrothermal vent, Cold seep, Biogeography

## Abstract

**Background:**

*Sclerolinum* (Annelida: Siboglinidae) is a genus of small, wiry deep-sea tubeworms that depend on an endosymbiosis with chemosynthetic bacteria for their nutrition, notable for their ability to colonise a multitude of reducing environments. Since the early 2000s, a *Sclerolinum* population has been known to inhabit sediment-hosted hydrothermal vents within the Bransfield Strait, Southern Ocean, and whilst remaining undescribed, it has been suggested to play an important ecological role in this ecosystem. Here, we show that the Southern Ocean *Sclerolinum* population is not a new species, but more remarkably in fact belongs to the species *S. contortum*, first described from an Arctic mud volcano located nearly 16,000 km away.

**Results:**

Our new data coupled with existing genetic studies extend the range of this species across both polar oceans and the Gulf of Mexico. Our analyses show that the populations of this species are structured on a regional scale, with greater genetic differentiation occurring between rather than within populations. Further details of the external morphology and tube structure of *S. contortum* are revealed through confocal and SEM imaging, and the ecology of this worm is discussed.

**Conclusions:**

These results shed further insight into the plasticity and adaptability of this siboglinid group to a range of reducing conditions, and into the levels of gene flow that occur between populations of the same species over a global extent.

**Electronic supplementary material:**

The online version of this article (doi:10.1186/s12862-015-0559-y) contains supplementary material, which is available to authorized users.

## Background

The vastness and inaccessibility of the deep sea has challenged scientists seeking to understand its diversity [1, 2]. A major area of this research concerns improving knowledge on the ranges of deep-sea species, which has become particularly pertinent in light of growing human impacts in this environment [3]. Molecular tools have been applied to this field and have revealed that certain deep-sea species with widespread distributions can exhibit similar morphology but considerable genetic differentiation between regions, and may thereby represent several closely related but geographically restricted species – so called ‘cryptic species’ [4–8]. Contrastingly, other studies have also revealed that some taxa can indeed be incredibly widespread, displaying distributions that can span both poles, i.e. bipolar. This pattern has been confirmed in bacteria and archaea [9, 10], in benthic foraminifera [11], deep-sea coral [12] and a lineage of the amphipod *Eurythenes gryllus* [8]. While there are problems with the use of molecular data to delimit species, the examination of genetic variation at multiple (both mitochondrial and nuclear) loci within an evolutionary context has become an important addition to our definition of a species alongside morphological, biological and ecological observations [13–15], as well as a critical tool in the investigation of species biogeography. Here we investigate the range and ecological adaptations of a deep-sea siboglinid tubeworm over near 16,000 km spanning from the Arctic to the Antarctic.

The family Siboglinidae is a monophyletic lineage of annelid worms comprised of the vestimentiferans, or giant tubeworms, the bone-eating genus *Osedax*, and two groups of slender tubeworms – *Sclerolinum* and the frenulates [16]. Siboglinidae is exceptional among the annelids due to this family’s almost complete reliance on endosymbiotic bacteria for nutrition, and the unusual morphology which its members have adopted for this specialism [17]. The majority of siboglinids (except for *Osedax* and a number of frenulates capable of oxidising methane) harbour sulphur-oxidising symbionts [18] and are characteristically long, often acting like a ‘bridge’ between a sulphidic substrate where their posterior end is located, and oxygenated seawater into which they extend their anterior end [19].

Although siboglinids are found within all of the world’s major oceans, the distribution and genetic structure of certain lineages is poorly constrained. Hydrothermal vent vestimentiferans endemic to the East Pacific Rise (EPR) are perhaps the best studied, where species such as *Riftia pachyptila* and *Tevnia jerichonana* show extensive ranges along the length of this mid ocean ridge system, while the degree of genetic differentiation between populations increases with distance [20, 21]. Vestimentiferans that can colonise seeps, whale and wood falls have the potential to be even more widely distributed. The genus *Escarpia* is found in a variety of reducing environments, and occupies several ocean basins with the three described species *Escarpia laminata*, *E. southwardae*, and *E. spicata* occurring in the Gulf of Mexico (GoM), West Africa, and in the eastern Pacific respectively. However, while there is high genetic similarity between the three species, geographical and hydrological barriers still appear to limit gene flow between them [22].

The genus *Sclerolinum*, which forms the sister clade to the vestimentiferans [23], also exhibits a widespread distribution. The seven formally described species are reported from the northeast Atlantic [24, 25], GoM and Caribbean [26, 27], and southeast Asia [28, 29], however there are also a number of known but not currently described *Sclerolinum* populations from Antarctica, Hawaii [30], the Sea of Okhotsk [31] and off Kushiro, Japan [32, 33], that extend the range of this genus even further. This little studied genus of small, wiry tubeworms have also been found to possesses peculiar organisation that has made it challenging to determine its position in relation to other siboglinids, have been shown to perform important ecological functions within deep-sea sediments, and is capable of colonising a multitude of reducing environments [25–27, 29, 30, 34].

Remarkable substrate choice and geographical range is demonstrated by just one *Sclerolinum* species, *S. contortum*. Initially described from soft sediments at Håkon Mosby Mud Volcano (HMMV) [25], this species was later also found to be residing in the nearby cold seeps of the Storegga Slide, Norwegian Sea [35, 36] as well as in diffuse flow areas of the Arctic vents of Loki’s Castle [37, 38]. Colonisation experiments in the northeast Atlantic have shown that in addition to soft sediments, *S. contortum* can inhabit wood, other decaying plant debris, as well as mineral substrates [39]. A population of *Sclerolinum contortum* notably also occurs within the cold seeps of the GoM, a distance of over 7600 km from the nearest northeast Atlantic population [27].

Considerable sampling of the deep waters around Antarctica in recent years has revealed this region to be much more diverse, and not as isolated as traditionally thought [40]. These exploration efforts have also shown that the Southern Ocean possesses a variety of deep-sea chemosynthetic habitats that include areas of high temperature and diffuse venting, cold seeps, and whale falls [41–44]. Hydrothermal activity is currently known to occur within the Bransfield Strait [41, 45], along the East Scotia Ridge [46], Pacific-Antarctic Ridge [47], Australian-Antarctic Ridge [48], and within Kemp Caldera [49], and to support unique vent ecosystems distinct from those of the main mid-ocean ridge systems [43].

**Fig. 1:**
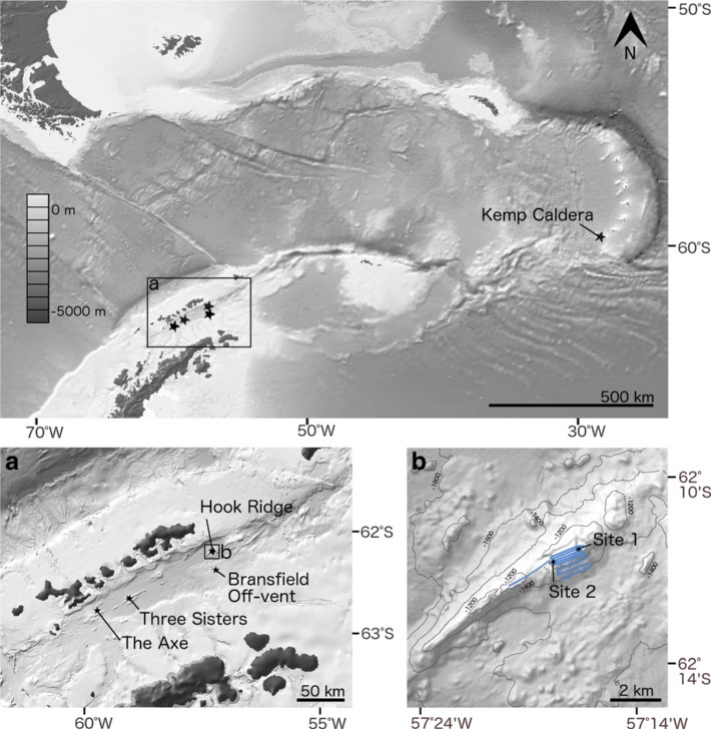
Southern Ocean sampling sites from which *Sclerolinum* sp. was collected. Box (**a**) shows the locations of JC55 Bransfield Strait sampling locations, and (**b**) shows detail of Hook Ridge sampling sites (Hook Ridge Site 1 and Hook Ridge Site 2), as well as the path traversed by the SHRIMP (in blue). Map created using GeoMapApp (http://www.geomapapp.org) using data from the Global Multi-Resolution Topography (GMRT) Synthesis [92]

Since 2001, *Sclerolinum* has been known to inhabit the sedimented hydrothermal vents of Hook Ridge, Bransfield Strait (Fig. 1) [41]. This population was recently suggested to play an important role in mediating the release of iron and manganese from sediments to the water column [34]. However while aspects of the habitat and function of this population have been investigated [30, 34], the morphology of these worms, their extent within the Southern Ocean, and how this population relates to other known *Sclerolinum* populations remain unknown. This study aims to provide a detailed description of the Antarctic *Sclerolinum* population, place it within a phylogenetic context and thereby establish its relationships to other *Sclerolinum* populations worldwide, and discern its extent and ecology within the Southern Ocean.

## Results

### Systematics

Phylum Annelida

Family Siboglinidae Caullery, 1914

Genus *Sclerolinum* Southward, 1961

***Sclerolinum contortum*Smirnov, 2000**

(Figs. 2, 3 and 4)

**Fig. 2:**
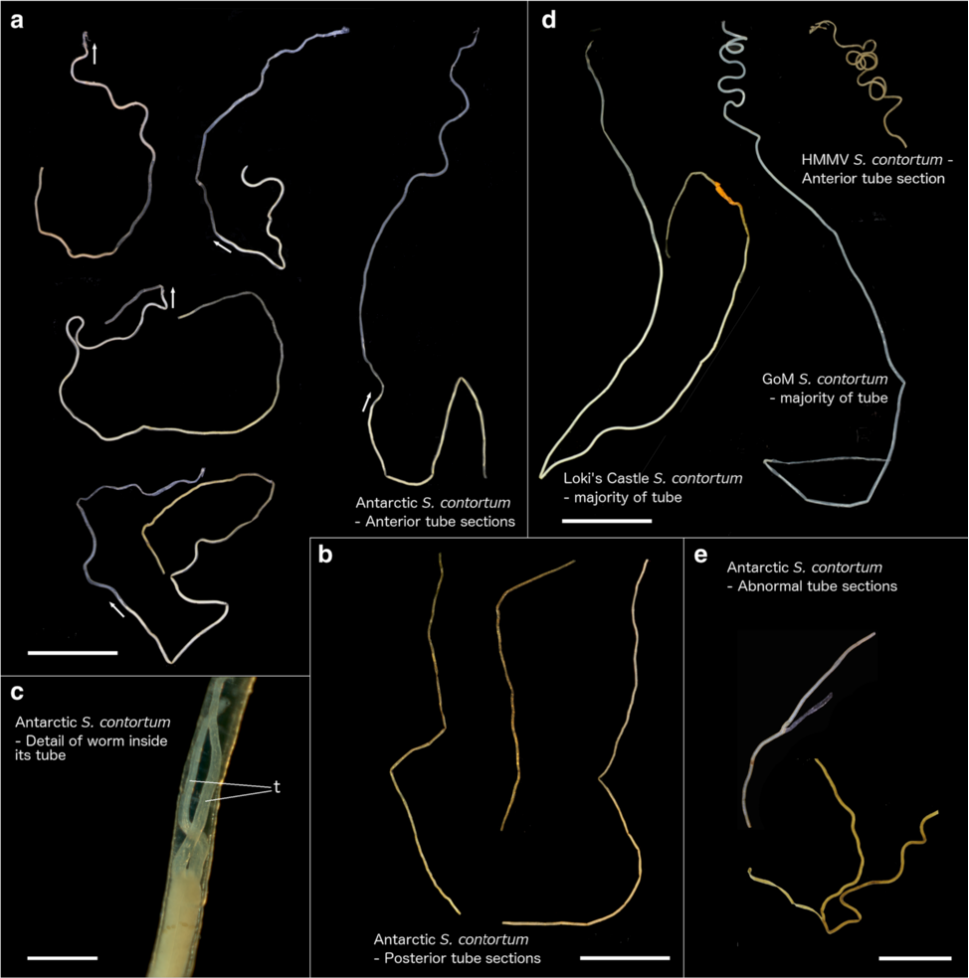
Broad morphology of *Sclerolinum contortum* tubes. **a** Antarctic *S. contortum* anterior tube sections, arrows indicate position and orientation of the worms’ heads. **b** Posterior sections of Antarctic *S. contortum* tubes. **c** Detail of tube with worm inside it, t – tentacles. **d** Tubes of *S. contortum* from Loki’s Castle, GoM and HMMV. **e** Antarctic *S. contortum* tube sections showing abnormalities. Scale bars for (**a**-**b**, **d**) are 10 mm, 400 μm for (**c**) and 5 mm for (**e**)

**Fig. 3:**
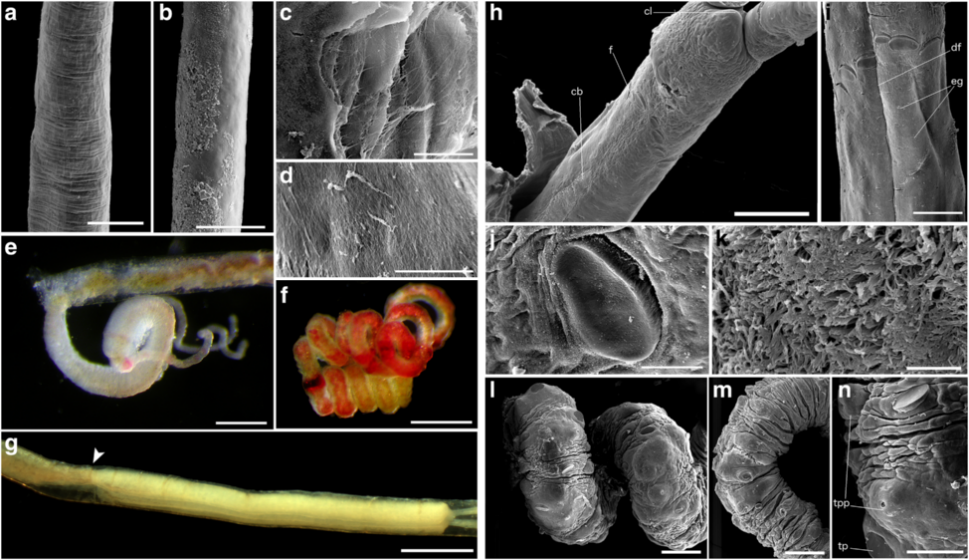
Details of tubes and tissues of Antarctic *Sclerolinum contortum*. **a** Anterior section of tube showing more pronounced transverse wrinkles and faint longitudinal wrinkles, scale bar is 200 μm. **b** Posterior section of tube showing a smooth tube wall with attached sediment, scale bar is 200 μm. **c** Detail of tube wall showing its multi-layered, fibrous structure, scale bar is 10 μm. **d** Detail of tube wall interior, scale bar is 5 μm. **e** Anterior portion of a live worm, scale bar is 300 μm. **f** Trunk tissue of a live worm, scale bar is 500 μm. **g** Anterior portion of a worm showing the transition between the forepart and trunk (arrow), scale bar is 500 μm. **h** The anterior of a worm in ventral view, scale bar is 100 μm. **i** Detail of the frenulum and surrounding gland openings, dorsal view. Scale bar is 50 μm. **j** Detail of a frenular plaque, scale bar is 10 μm. **k** Detail of the ventral ciliary field, scale bar is 5 μm. **l**-**n** Trunk tissues of a worm, scale bars in (**l**-**m**) are 100 μm and 50 μm in (**n**). cb – ciliated band; cl – cephalic lobe; df – dorsal furrow; eg – epidermal glands; f – frenulum; tp – trunk plaque; tpp – trunk papillae

**Fig. 4:**
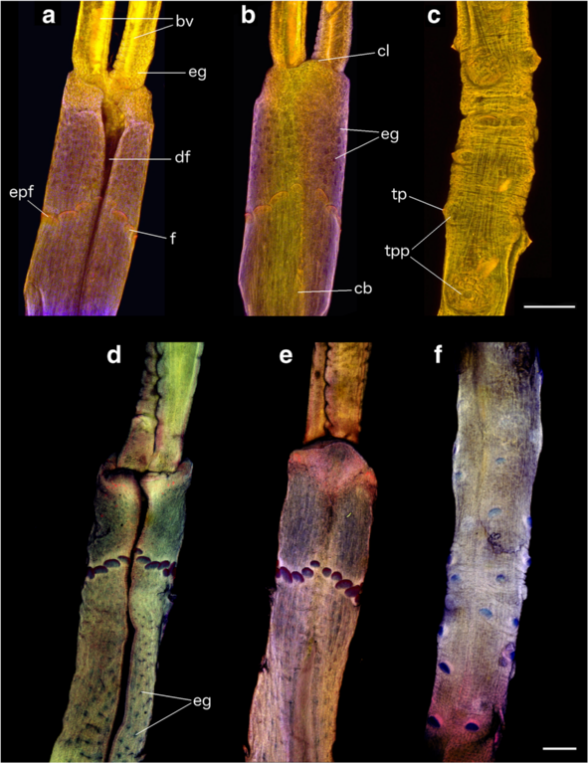
Confocal laser scanning microscopy images of *Sclerolinum contortum*. **a**-**c** show Antarctic *S. contortum*, and (**d**-**f**) show *S. contortum* from Loki’s Castle. **a**, **d** anterior section, dorsal view; (**b**, **e**) anterior section, ventral view; (**c**, **f**) – portion of trunk. All scale bars are 100 μm. bv – blood vessels; cb – ciliated band; cl – cephalic lobe; df – dorsal furrow; eg – epidermal glands; epf – elongated plaque of frenulum; f – frenulum; tp – trunk plaque; tpp – trunk papillae

### Material examined

Southern Ocean, *Hook Ridge Site 1*, 62.1969°S 57.2975°W, 1174 m depth: JC55_19 (RRS *James Cook* operation no.), 15 tube fragments. JC55_19, tubes attached to sampling gear (non-quantitative), 234 tube fragments [NHMUK 2015.1140-1146]. JC55_20, five tube fragments [NHMUK 2015.1153-1155]. JC55_21, 1 worm fragment with forepart, seven tube fragments [NHMUK 2015.1147-1152]. JC55_25, 29 worm fragments with forepart, 302 additional tube fragments [NHMUK 2015.1156-1157, 1188–1189 ( subset of examined material )]. *Hook Ridge Site 2*, 62.1924°S 57.2783°W, 1054 m depth: JC55_30, eight tube fragments attached to sampling gear [NHMUK 2015.1158].

### Description

Anterior extremity of tubes pale white in colour, thin walled (2 to 7 μm) and flattened. Posteriorly, wall thickness increases (to maximum of 28 μm) and tubes generally exhibit several smooth bends (Fig. 2a). Majority of tube is pale brown/green in colour (Fig. 2a-b), flexible and elastic, often possessing closely but irregularly spaced transverse wrinkles as well as faint longitudinal wrinkles on its outer surface (Fig. 3a), occasional microbial filament and rust patches are also present on outer tube surfaces. Tube walls are multi-layered, comprised of superimposed fibrous sheets in which fibres show an overall disorganised arrangement, inner tube surface shows a similar texture (Fig. 3c-d). Towards posterior extremity, tubes are increasingly thin walled and collapsed, outer tube wall generally smooth but with patches of attached sediment grains (Fig. 3b). Tube diameter ranges from 0.22 to 0.30 mm, longest tube fragment measured 155 mm. Several tubes exhibit branch-like abnormalities (Fig. 2e), a subset of Hook Ridge Site 1 tubes were very dark brown to black in colour (similar to tubes from Kemp Caldera, see later (Fig. 6b)).

Longest animal measured 52 mm in length (from tip of the cephalic lobe) but all were incomplete with posterior extremity missing, therefore opisthosomal characters could not be elucidated. Animals are bright red when alive, this colouration being most pronounced in trunk tissues (Fig. 3e-f). Two tentacles (Fig. 2c) often slightly different in length in individuals, tentacle lengths overall varied greatly between measured worms, from 0.83 to 3.00 mm. Tentacles smooth or occasionally wrinkled on inner surfaces, longitudinal blood vessels visible within them (Fig. 4a-b). Dense epidermal glands occur around the base of tentacles, which are more scattered distally (Fig. 4a-b). Diameter of forepart ranges between 0.15 to 0.23 mm. Cephalic lobe had a small, rounded triangular tip 55 to 75 μm in length that protrudes from forepart (Figs. 3h and 4b). Dorsal furrow deep and wide, extending from base of tentacles (Figs. 3i and 4a). Frenulum positioned 0.13 to 0.37 μm from tip of cephalic lobe. Region surrounding frenulum shows dense covering of glands, present on both dorsal and ventral surfaces. Frenulum comprised of 9–19 oval to elongated plaques measuring 14 to 46 μm in diameter (Fig. 3j), occurring as a slightly sparse or dense row. Frenular plaques occur dorsolaterally and ventrally with middle ventral plaque often missing, plaques in middle ventral and middle dorsal areas often smaller (Figs. 3h-i and 4a-b). Densely ciliated band present posterior to frenulum on ventral side of animal, that widens with increasing distance from frenulum (Figs. 3h, k and 4b). Region posterior to frenulum and around the ciliated band contains scattered glands, visible as slits in SEM images (Fig. 3i).

Transition between ending of dorsal furrow and beginning of a narrower, highly wrinkled and densely papillated trunk region clearly distinguished anterior and posterior zones of Antarctic *S. contortum* animals (Fig. 3g), with this forepart region measuring 1.7 to 4.8 μm in length from the tip of the cephalic lobe to the beginning of the trunk. The trunk (Figs. 3l-n and 4c) comprised much of length of animals and was characterised by the presence of scattered oval plaques positioned on top of papillae (Fig. 3l-n). Large papillae without plaques, possibly openings of pyriform glands, also present in trunk region (Fig. 3n).

### Remarks

The conspecificity of Antarctic *Sclerolinum* with HMMV, Loki’s Castle and GoM *S. contortum* is strongly supported by genetic data (see later). *S. contortum* (from HMMV) was originally distinguished from all other species in this genus based on its long opisthosoma with few segments, and a strongly twisted anterior tube region [25]. The anterior regions of tubes from the Antarctic however lack the characteristic prominent, knot-like contortions that lend *S. contortum* its name, being instead only faintly wavy. These contortions are also absent in some of the Loki’s Castle specimens (Fig. 2d). In addition, the GoM population shows that *S. contortum* opisthosoma can be longer than those of *Sclerolinum magdalenae* [26] and possess a similar number of segments. Hence we do not believe the wavy nature of the tube and the length of the ophisthosome are useful characters to delineate species. *S. magdalenae* also has a similar frenulum to *S. contortum*, making these two species difficult to distinguish based on currently used characters. This raises the question of whether *S. magdalenae* may be the same species as *S. contortum* and thereby represent yet a further example of the wide range of this species; molecular analyses on *S. magdalenae* would be needed to clarify this.

Antarctic *S. contortum* most closely resembles the HMMV population in terms of size (diameter of tube and animal, forepart length, frenular plaque size and number; Additional file 1: Table S1). Although animals from the various populations show broad similarity (Fig. 4) [25, 27], this species is known to show pronounced morphological plasticity of its soft tissues [27] and incorporating data from the Antarctic and Loki’s Castle extends the ranges of taxonomic characters for this species even further (Additional file 1: Table S1).

Ultrastructurally, tubes do not vary much between the Arctic, GoM and Antarctic populations and all exhibit both transverse and longitudinal wrinkles, while the tube abnormalities pictured in Fig. 2e are similar to those recorded for *Sclerolinum brattstromi*, *Siboglinum ekmani* and *Siboglinum fiordicum* [50].

### Ecology

**Fig. 5:**
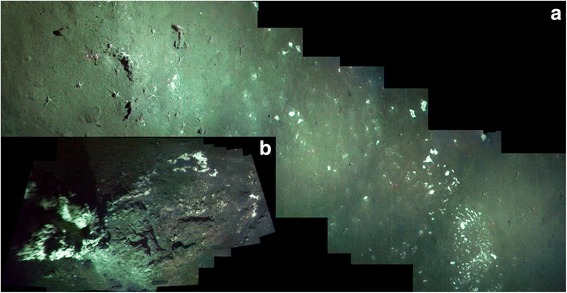
SHRIMP images of Hook Ridge, Southern Ocean. Images were taken near to where megacore samples containing the highest density of *Sclerolinum contortum* were collected (maximum of 20 m distance). **a** Soft sediment with bacterial mats, (**b**) vent chimney of unknown activity with associated bacterial mats

Living animals were most abundant at Hook Ridge Site 1, where *S. contortum* has been reported at high densities (up to 800 individuals m^−2^ [30]), and tube fragments with tissue were also abundant at Hook Ridge Site 2. However, the distribution of *S. contortum* at Hook Ridge appears to be patchy as one of the megacore samples contained only a single specimen with a head, while another contained 71 individuals [51]. Worm specimens were not visible within the megacore tubes until the samples were processed, suggesting that the majority of the tubes were buried within sediments. The posterior ends of the tubes were recorded as occurring at 15 cm depth by Sahling et al. [30], where temperatures are approximately 20 °C and hydrogen sulphide concentrations reach 150 μm L^−1^, increasing at greater depths [34]. No temperature anomalies were observed in any sediments during sampling in 2011 [34]. A fully oxic water column, and oxygen penetration to depths of 2–5 cm into the sediment [34] require little of the tubes to project above the sediment. *Sclerolinum* is not reported from parts of the Hook Ridge where temperatures reach 49 °C and siliceous crusts form over the sediments [52]. SHRIMP (Seafloor High Resolution Imaging Platform) images (Fig. 5) in the vicinity of Hook Ridge Site 1 give an indication of the habitat of this species. Diffuse hydrothermal flow in this area is evidenced through the presence of what are inferred to be patchy bacterial mats (white patches in Fig. 5a). These mats also occurred around vent chimneys present at this site (Fig. 5b) the activity of which is unknown but again no temperature anomaly was observed within what appeared to be shimmering water emanating from the chimney structure [45].

***Sclerolinum*sp. Southward, 1961**

(Fig. 6)

**Fig. 6:**
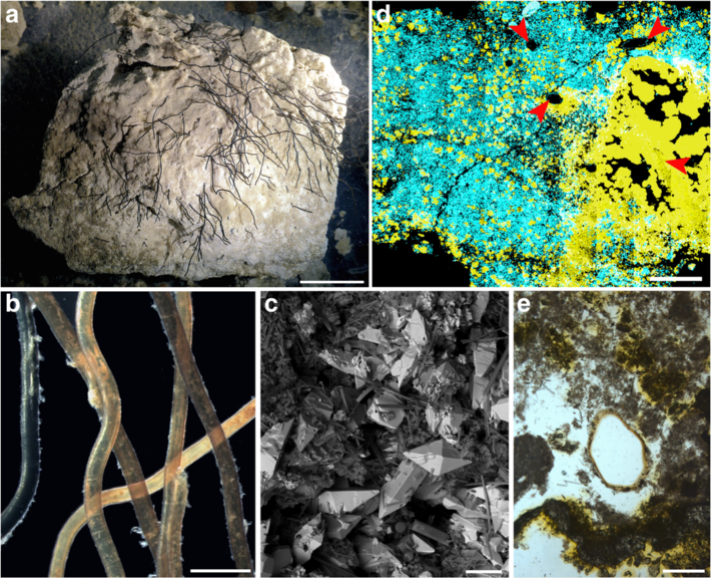
Sulphurous lump with embedded *Sclerolinum* tubes collected from Kemp Caldera. **a** Sulphurous lump with embedded *Sclerolinum* tubes collected from Kemp Caldera, scale bar is 30 mm. **b** Detail of the tubes embedded in the sulphurous lump pictured in (**a**), scale bar is 1 mm. **c** SEM image of the surface of subsample of the sulphurous lump, scale bar is 50 μm. The bright crystals in this image showed a large sulphur peak when examined using energy dispersive x-ray spectroscopy (EDS). **d** EDS elemental map of sulphur lump subsample, yellow colours highlight the distribution of sulphur, blue of silicon. Red arrows show *Sclerolinum* tubes in section, scale bar is 2 mm. **e** Detail of *Sclerolinum* tube section that is embedded within the sulphurous lump, scale bar is 200 μm

### Material examined

*Kemp Caldera*, 59.6948°S 28.35°W, 1432 m depth: JC55_106, lump of sulphurous material attached to sampling gear with embedded tubes. Ninety-one tube fragments removed from lump, and 4 possible tissue fragments removed from tubes and preserved separately [NHMUK 2015.1159-1166].

### Description

Tubes clustered and tightly embedded into upper surface of sulphurous material (Fig. 6a), 0.23–0.34 mm in diameter (*n* = 10) and with a tube wall thickness of approximately 30 μm. Wavy to near straight in appearance. Outer tube surfaces exhibit prominent, irregular transverse wrinkles and faint longitudinal wrinkles (Fig. 6b). Tube walls are multi-layered and fibrous, and in some cases have a very rough appearance due to fragmentation of outer tube layers. SEM and EDS (energy dispersive x-ray spectroscopy) of the surface of the sulphurous lump shows large crystalline sulphur grains within a silica matrix (Fig. 6c). When mapped in thin section, sulphur and silica show some zonation but also many sulphur grains incorporated into areas of silica (Fig. 6d). *Sclerolinum* tubes are rooted beneath the surface of the sulphur lump, Fig. 6e shows detail of the sulphurous material with one of the embedded tubes.

### Remarks

These tubes show very similar overall and detailed morphology to those made by *Sclerolinum contortum* from Hook Ridge, and it is very likely that they were therefore made by this species, however as no intact animals were found (unidentifiable tissue was present) it was not possible to confirm this. The significant lengths of the tubes (Fig. 6a) suggest that the colony may have reached maturity, however the absence of good quality animal tissue, the inability to DNA sequence tube contents, and the rough appearance of some of the tube walls suggest that the colony had started degrading and that conditions may have become unfavourable for *Sclerolinum*. The sulphur chunk also demonstrates a pathway through which *Sclerolinum* tubes may fossilise, preserved as *Sclerolinum* tube-shaped voids within its matrix (Fig. 6d).

### Ecology

Areas of diffuse venting within Kemp Caldera would be favourable habitats for *Sclerolinum*, however the occurrence of these animals within such a highly acidic environment, within a substrate composed largely of sulphur has not previously been observed.

### Phylogeny and genetic diversity of *S. contortum*

**Fig. 7:**
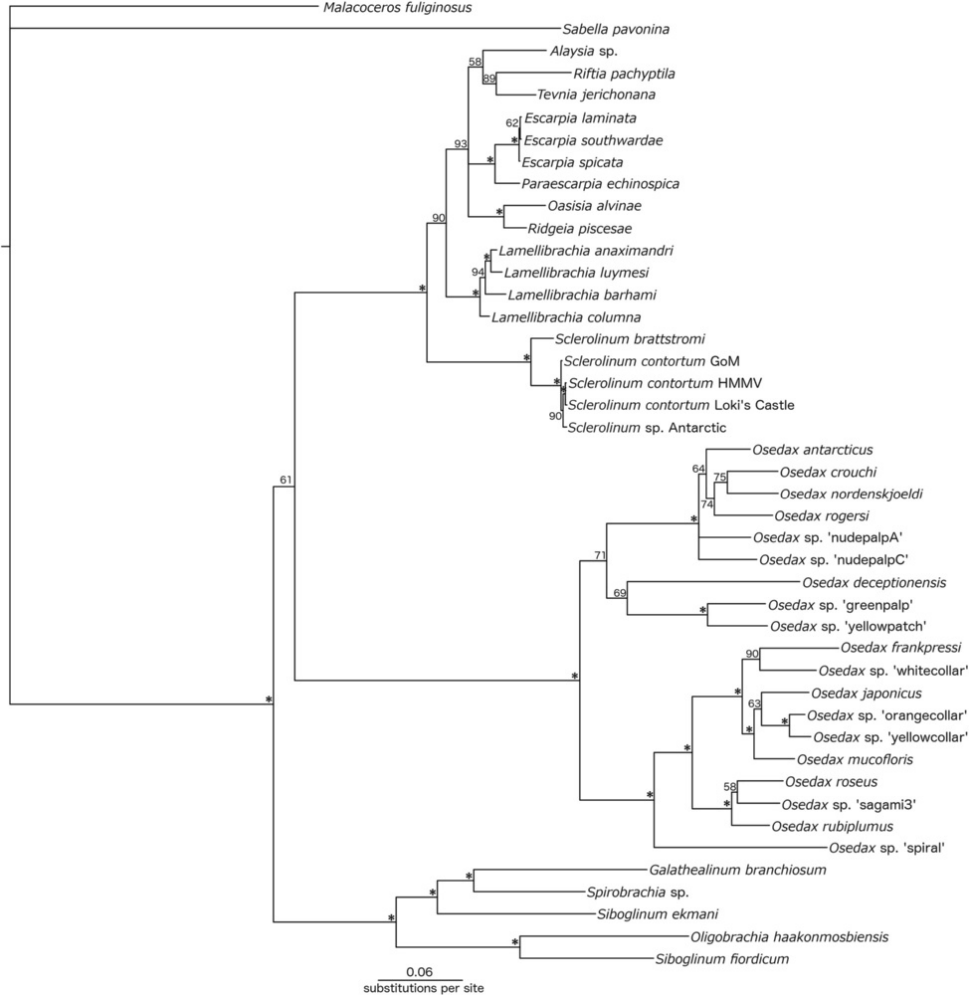
Phylogeny of the annelid family Siboglinidae. This analysis was performed using a Bayesian approach and a combined dataset of the three genes COI, 16S and 18S. The phylogeny is a 50 % consensus tree, in which numbers represent posterior probability values out of 100, and branches marked with an asterisk (*) indicate posterior probabilities equal to or greater than 95

The three combined molecular analysis runs for the family Siboglinidae converged on the same tree topology and near identical posterior probability values (maximum variation of 4 %). The 50 % majority rule consensus tree (Fig. 7) indicated overall strong branch support for the monophyly of the major clades of Vestimentifera, *Sclerolinum*, *Osedax* and Frenulata. Antarctic *Sclerolinum* falls within a clade comprised of *S. contortum* from GoM and the Arctic, where GoM worms form a sister group to *S. contortum* from the Arctic and Antarctic *Sclerolinum*, with strong branch support. However, support for the sister relationship between Antarctic *Sclerolinum* and Arctic *S. contortum* is weaker. 18S was identical between *Sclerolinum brattstromi,* HMMV *S. contortum*, and Antarctic *Sclerolinum*, whereas for 16S, one change (a transversion) was detected between Antarctic *Sclerolinum*, and *S. contortum* from GoM, Loki’s Castle and HMMV (*S. brattstromi* 16S was very different). COI K2P (Kimura 2 Parameter) and ‘*p*’ distances within the *Sclerolinum* clade varied from 0 % between *S. contortum* populations, to 1.4 % between *S. contortum* and Antarctic *Sclerolinum*, and were almost an order of magnitude greater between these taxa and *S. brattstromi* where the minimum distance detected was 8.8 % (Additional file 2: Table S2). Within the *S. contortum* clade, the lowest genetic distances occurred between Loki’s Castle and HMMV populations, and the greatest between the Arctic and Antarctic populations (Additional file 2: Table S2).

**Fig. 8:**
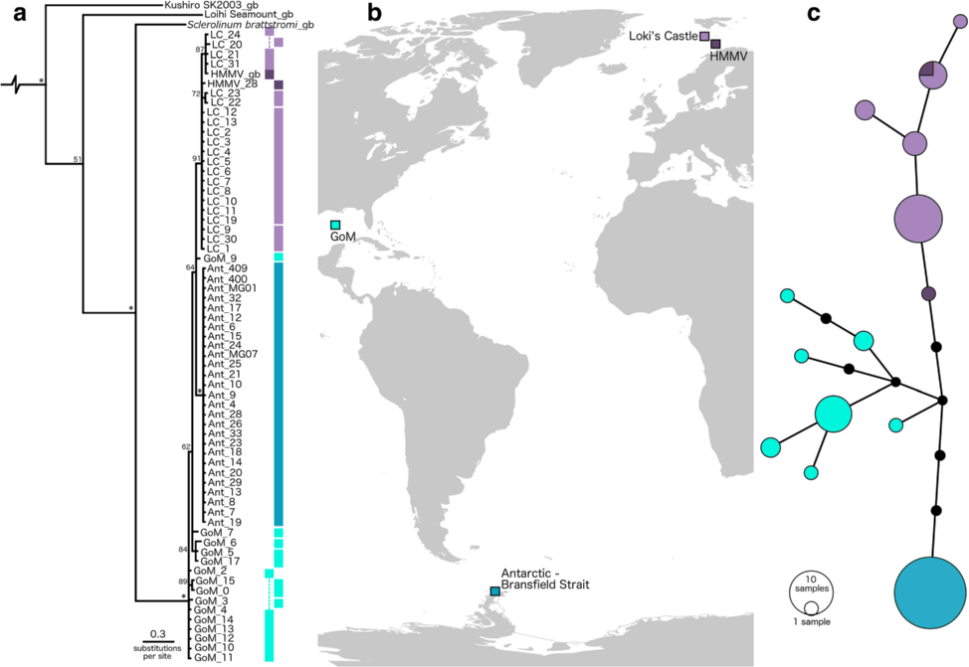
Results of phylogenetic and haplotype network analyses for *Sclerolinum contortum*. **a** Phylogeny of *S. contortum* individuals using the COI gene with vertical bars representing haplotype groups, coloured according to population location; HMMV -dark purple, Loki’s Castle (prefix LC) – light purple, GoM – light blue, and the Antarctic (prefix Ant) – dark blue. The siboglinids *Riftia pachyptila* and *Siboglinum ekmani* were used as outgroups (not shown), and sequences obtained from NCBI GenBank have the suffix ‘_gb’ (see Additional file 5: Table S4 for accession numbers). **b** Map of the Atlantic and part of the Southern Ocean showing the locations of the four *S. contortum* populations used in this study, world map source: Wikipedia. (https://en.wikipedia.org/wiki/Contemporary_history#/media/File:WorldMap.svg). **c** Haplotype network constructed using the gene COI, sequenced from animals from four different *S. contortum* populations. Gaps were treated as missing data, and the connection limit was set to 95 %. Each line represents one change, and black dots represent missing haplotypes. Haplotype network was drawn using PopART (http://popart.otago.ac.nz)

The phylogenetic and haplotype analyses based on 65 *S. contortum* COI sequences showed 14 distinct haplotypes (Fig. 8; Table 1; maximum variation of 3 % for posterior probability values within the phylogenetic analysis). The number of haplotypes within the Arctic and GoM populations were greater than within the Antarctic population, in which all 27 individuals form a single haplotype despite having the largest sample size. An HMMV individual fell within the same haplotype as Loki’s Castle worms, and as genetic distances were lowest between these two populations (Additional file 2: Table S2), HMMV and Loki’s Castle sequences were henceforth pooled into a single Arctic population. Nucleotide diversity (π) and mean K2P distances within populations were on the whole low, and a non-synonymous substitution was found within the Arctic population (Table 1). The results of the analysis of molecular variance (AMOVA) (Table 2) show that the largest percentage of variation occurs between the three regional populations, which also resulted in a large *F*_*ST*_ value, whereas within population variation is considerably lower. Pairwise *F*_*ST*_ values are high, significant, and increase with distance between populations, being greatest between the Antarctic and Arctic populations and lowest between the Arctic and GoM populations.

**Table 1:** Measures of COI sequence variation within *S. contortum* populations

Locality	*N*	No. of haplo-types	Haplotype diversity (h)	Nucleotide diversity (π)	No. of polymorphic sites (S)	No. of synonymous/non-synonymous substitutions	Mean K2P distance (%)
Arctic	23	6	0.700 ± 0.088	0.002 ± 0.0004	5	4/1	0.2
GoM	15	7	0.781 ± 0.102	0.004 ± 0.0008	10	10/0	0.4
Antarctic	27	1	0	0	0	0	0
Total	65	14					

N - sample size, standard deviations are given for h and π

**Table 2:** Results of the AMOVA for the various *S. contortum* populations

Source of variation	Degrees of freedom	Sum of Squares	Variance components	Percentage of variation
Among populations	2	102.573	2.41055 Va	84.5
Within populations	62	27.424	0.44232 Vb	15.5
Total	64	129.997	2.85287	
Fixation index (*F*_ST_)	0.84496			
Pairwise *F*_ST_ values				
	Arctic	GoM		
GoM	**0.7211**			
Antarctic	**0.9095**	**0.8621**		

F_ST_ values in bold are significant (*p* < 0.05)

## Discussion

### First record of a bipolar siboglinid: geographic and genetic patterns

Our data strongly supports the notion that *Sclerolinum contortum* is a bipolar species, with records that span almost 16,000 km from the Arctic to the Antarctic and making it the only siboglinid for which such a range has been observed. Our combined phylogenetic analysis using extended molecular data for the *Sclerolinum* genus demonstrates high levels of similarity of three barcoding genes COI, 16S and 18S between Antarctic *Sclerolinum* and *S. contortum* from the Arctic and GoM, and clearly distinguishes another *Sclerolinum* species (*Sclerolinum brattstromi*) from this group (Fig. 7). In addition, the mitochondrial marker COI also differentiates an additional two *Sclerolinum* species from the *S. contortum* clade (Kushiro SK2003 and Loihi Seamount; Fig. 8). COI genetic distances are more than 6 times greater between *S. contortum* (including the Antarctic population) and *S. brattstromi* compared to within the former clade (Additional file 2: Table S2), in which divergence is well below 3 %, the generally accepted threshold for delimiting species [53, 54]. The morphology of Antarctic *Sclerolinum* also generally fits within the variation observed for *S. contortum* from other areas, most closely resembling the soft tissue morphology of the most distant population, from HMMV (Additional file 1: Table S1). Classifying Antarctic *Sclerolinum* as *S. contortum* despite the great distances between populations highlights the important taxonomic observation that annelids with very similar morphology and DNA can be spread over vast geographical areas, with their distributions controlled by habitat availability and local ecology.

Bipolarity has so far been observed in only a handful of deep-sea organisms, but demonstrates that steep temperature gradients and limited water exchange between the Southern and surrounding oceans have not completely restricted the spread of deep-sea fauna across these barriers [40]. Southern Ocean vent sites such as the East Scotia Ridge differ from sites on Mid-Atlantic Ridge and East Pacific Rise in that fauna such as vestimentiferan and alvinellid polychaetes, vesicomyid clams, bathymodiolid mussels, and alvinocaridid shrimp are absent [43]. However the ability of *S. contortum* to have migrated across Southern Ocean dispersal barriers suggests that the absence of vestimentiferans at Antarctic vent sites may not be the result of historical dispersal limitation (vicariance). The extensive, bipolar nature of this deep-sea chemosynthetic tubeworm also accentuates that being widespread in the deep-sea is a real and common pattern, with examples supported by molecular data emerging from a variety of additional taxa in recent years [8, 55–58].

Although our data support *S. contortum* conspecificity across the Arctic, GoM and Antarctic, at a population level there is evidence that distance is a barrier to gene flow. While mixing appears to occur between the HMMV and Loki’s Castle populations that are separated by approximately 270 km (Fig. 8), this does not seem to be the case between the Arctic, GoM and Antarctic. Though these three regional populations show very high genetic similarity, the structure presented by the COI haplotype network (Fig. 8), and the *F*_ST_ values obtained for population pairs (Table 2), suggest that geographic distance does present a barrier to gene flow for this species. This is largely consistent with research into the connectivity of hydrothermal vent vestimentiferans on the EPR, where for both *Riftia pachyptila* and *Tevnia jerichonana* there appears to be little gene flow between the most distant populations of these species [20, 21]. Pairwise *F*_ST_ values between the most distant populations of these two species are similar to those reported for *S. contortum* in this study, however it is obvious that many populations of *S. contortum* are likely to exist between those sampled in this study, and sampling gaps have been found to inflate *F*_ST_ [59].

High genetic correspondence with lack of gene flow over large distances is also characteristic of *Escarpia* spp., species of which show high levels of similarity in the mitochondrial genes 16S, COI and cytochrome *b*, but can be differentiated on their morphology, as well as by using a nuclear gene (haemoglobin subunit B2 intron) and microsatellite markers [22]. Our interpretation of geographically distant *Sclerolinum* populations belonging to one species contrasts with the division of *Escarpia* into three separate species despite their genetic similarity, however we believe our classification to be justified based on the reasons outlined above, and recommend greater caution in describing genetically-similar but geographically distant populations of siboglinids as new species based on morphology.

Despite the evidence for low gene flow at regional scales, *Sclerolinum contortum* has managed to spread to both poles as well as subtropical latitudes, and the question remains as to how this was achieved. Nothing is presently known of the larvae of *Sclerolinum*, but when the larvae of the vestimentiferan *Riftia pachyptila* are considered, which can disperse 100 km along the EPR ridge axis [60], it is unlikely that *S. contortum* larvae travelled the ~10,000 km between the GoM and Hook Ridge in a single journey. As *S. contortum* appears to be capable of colonising a large range of substrates, dispersal over wide areas through the use of a variety of chemosynthetic habitats as ‘stepping stones’ [61, 62] might be the most plausible explanation for this species. Such a hypothesis may be supported by our results which show that there is greater genetic similarity between the spatially closer Arctic and GoM populations, and GoM and Antarctic populations, than there is between the two polar populations (Arctic and Antarctic; Table 2). However, the presently known number of *S. contortum* populations is too low to conduct a test for the above scenario, therefore whether this is the best model cannot be resolved at present. Stepping-stone dispersal would suggest that *S. contortum* is more widespread than currently supposed, which does appear to be the case in the Antarctic. The large mass of tubes recovered from Kemp Caldera suggests that *S. contortum* populations come and go, taking advantage of reducing conditions where they are encountered and dying out when these temporary oases dry up.

Such a lifestyle may also explain the low COI haplotype diversity observed within the Antarctic population in comparison to the Arctic and GoM worms used for this study. The Antarctic population may be demonstrating the effects either of a founder event or bottleneck [63], where a founder effect may arise as a result of a number of opportunistic *S. contortum* individuals finding suitable conditions and settling at Hook Ridge, and persisting in the sustained diffuse hydrothermal flow at this site. However, the ephemeral nature of hydrothermal circulation within the Bransfield Strait [45], and repeated glacial-interglacial events affecting the Southern Ocean mean that it may also be plausible for the Antarctic *S. contortum* population to have undergone a bottleneck (loss of genetic diversity following a population crash) [64]. Evidence of a genetic bottleneck linked to glacial cycles has been detected for a number of Antarctic species (see [65] for a review), and ultimately more samples from a wider area of the Southern Ocean would be required to test this in *S. contortum. Sclerolinum* has also been shown to be capable of asexual reproduction via breaking and regenerating missing ends [66, 67], which may also account for the low genetic diversity of the Antarctic population.

There is currently no fossil record for *Sclerolinum*. As well as demonstrating a pathway through which *Sclerolinum* tubes may become preserved in the fossil record, this study shows that any future reports of *Sclerolinum* fossil discovery should be mindful of the following: fossils found in a range of ancient chemosynthetic environments, from very distant parts of the world, and exhibiting varying degrees of tube contortion may belong to the same species. Recent reports of Cretaceous *Osedax* fossils [68] imply that Siboglinidae has more ancient origins than indicated by molecular clock estimates [69–71], suggesting that the widespread distribution, morphological and habitat plasticity exhibited by *Sclerolinum* may have contributed to the survival of this genus over long evolutionary timescales too [72].

### Natural history of *S. contortum* in the Southern Ocean

We have shown that *S. contortum* can exhibit even greater morphological plasticity than was previously noted for this species by Eichinger et al. [27]. Much of this plasticity is in the tubes constructed by this worm after which the species is named. Tube morphology may be a condition that is dictated by environmental factors, as has previously been shown for the highly plastic tubes built by the vent dwelling vestimentiferan *Ridgeia piscesae* [73]. Environmental factors can also influence the physiology of these worms, thereby affecting their genetic diversity [74]. While environmental parameters were not measured by the present study, we speculate that contortion of the anterior of *S. contortum* tubes increases their surface area to volume ratio, thus improving the efficiency of oxygen uptake and may therefore result from settlement in lower oxygen conditions.

The obvious morphological plasticity of *Sclerolinum contortum* is matched by its remarkable ecological and habitat plasticity. With our new data from the Antarctic we can now show that it is able to colonise a vast range of chemosynthetic habitats including high-temperature acidic white smoker vent fields, low-temperature sedimented diffuse vent fields, hydrocarbon cold seeps and mud volcanoes. Chemosynthetic invertebrates have been likened to terrestrial weeds [75] in virtue of their ability to colonise ephemeral/disturbed environments, as well as their effective dispersal, rapid growth rates, and early reproduction [76, 77], and in this sense, we can also think of *S. contortum* as a ‘chemosynthetic weed’ due to its ability to quickly populate a wide range of sulphur-rich habitats and spread over great distances.

Weedy species can have a dramatic influence on the environments they colonise. Their impacts are well-documented particularly in reference to terrestrial non-native species, and have demonstrated the ability of weedy species to have pronounced ecosystem, community and population-level effects [78, 79]. Supporting the concept of the *Sclerolinum* weed is the observation that the species can have a considerable influence on the biogeochemistry of the sediment at the sediment-hosted Bransfield hydrothermal vents [34]. Along with the maldanid *Nicomache lokii*, *S. contortum* forms a complex three-dimensional habitat for free-living invertebrates at Loki’s Castle [38], as well as in the Nyegga seep area of the Storegga Slide where filamentous bacteria cover *S. contortum* tube surfaces, thereby also providing substrate and food for associated organisms [35]. *S. contortum* therefore represents an important keystone species within the range of reducing environments it inhabits.

## Conclusions

Since their initial discovery alongside hydrothermal vent chimneys in the late 1970s, siboglinid worms have continued to surprise and amaze with their unusual adaptations to a mode of life in the deep sea dependant solely on endosymbionts. By investigating in detail the DNA, morphology and a novel inhabiting substrate of the very poorly studied *Sclerolinum* genus, the present study has found that they too conform to this pattern, by possessing extraordinary morphological and ecological plasticity that has allowed them to occupy a remarkable range that spans across all of the world’s oceans. However, fundamental knowledge of the biology of these worms is still lacking - there is presently no information on *Sclerolinum* reproduction, larvae and their dispersal, and symbionts from the range of chemosynthetic environments which this genus occupies. We therefore suggest these areas as potential directions for future research into this group.

## Methods

### Sample collection

Antarctic sample collection was conducted on board RRS *James Cook* expedition JC55 during January-February 2011 (Table 3), during which *Sclerolinum* was collected from two locations: Hook Ridge, Bransfield Strait, and Kemp Caldera. At Hook Ridge, venting occurs through sediment as low temperature discharge of phase-separated fluids that are highly diluted by seawater [45]. At Kemp Caldera, both hot and diffuse venting has been found that is characterised by unusual, highly acidic and sulphidic fluid composition. At a site named ‘Winter Palace’, crumbly chimneys release white smoker-type hydrothermal fluids up to 212 °C, while at ‘Great Wall’ a seafloor fissure releases low temperature diffuse fluids from which sulphur-rich minerals precipitate [80] [Copley et al. *in prep*.].

**Table 3:** Collection details of Siboglinidae specimens examined within this study

Locality	Taxon	Site	Latitude	Longitude	Depth (m)	No. of tube fragments*
Antarctic	*Sclerolinum* sp.	Hook Ridge Site 1	−62.1969	−57.2975	1174	686*
		Hook Ridge Site 2	−62.1924	−57.2783	1054	87*
		Kemp Caldera	−59.6948	−28.35	1432	95*
Arctic	*S. contortum*	Loki’s Castle CG2009	73.5662	8.1585	2357	33*
		Loki’s Castle CG2008	73.5662–73.5683	8.1585–8.1563	-	8*
		HMMV CG2010	71.9975–71.9999	14.7329–14.7316	1262	1*
		HMMV VICKING 2006	72.0013	14.7225	1270	50+
GoM	*S. contortum*	Walker Ridge WR269	26.6833	−91.65	1954	21*

Asterisk (*) denotes samples within which a subset of the tube fragments contained animal tissues

Samples were obtained using a Bowers & Connelly megacorer fitted with multiple 10 cm-diameter polycarbonate core tubes. *Sclerolinum* sp. tubes containing animal tissues and empty *Sclerolinum* sp. tubes were collected from two Hook Ridge sites, Hook Ridge Site 1 and Hook Ridge Site 2 (Fig. 1; Table 3). *Sclerolinum* sp. tubes from Kemp Caldera were acquired using a gravity corer, to which a sulphurous lump containing embedded tubes had become attached. Possible Siboglinidae tube fragments were collected from The Axe and Bransfield Off-vent, the latter comprising a non-active site located approximately 21 km south of the Hook Ridge sites. Samples were preserved in 80 % ethanol or 6 % formalin on board the ship. SHRIMP was used to visualise the seabed within a 20 m radius of Hook Ridge Site 1. *S. contortum* specimens from Loki’s Castle and HMMV, Arctic Ocean, and the GoM (Additional file 3: Methods supplement) were used for morphological and genetic comparisons with Antarctic *Sclerolinum* sp. (Table 3).

### Morphological and compositional analyses

Taxonomic characters were measured in 10 Antarctic and 10 Loki’s Castle worms. Unfortunately no complete animals were found, therefore only characters of the anterior and trunk regions of the worms were recorded. Tubes were either cut around sections of the worms, or they were visualised through their tubes using a ZEISS Discovery V.20 stereomicroscope. Measurements were performed using ZEISS AxioVision digital processing software as well as ImageJ (version 1.46r). To visualise taxonomic characters more clearly, sections of Antarctic and Loki’s Castle worms were cut out of their tubes, and imaged using laser-induced autofluorescence within a Nikon A1-Si Confocal microscope at the Natural History Museum, UK (NHM), operated in spectral imaging mode. In addition, the forepart and trunk regions of a *Sclerolinum* sp. worm fragment from the Antarctic were critical-point dried, coated in gold-paladium, and imaged using a secondary electron detector within a FEI Quanta 650 FEG-ESEM (NHM).

A subsection of the sulphurous lump with embedded *Sclerolinum* sp. tubes (recovered from Kemp Caldera, Southern Ocean) was viewed within a LEO 1455VP SEM (at the NHM), and point EDS spectra were obtained from its surface within the same SEM. The subsection was then prepared into a polished thin section and its elemental composition was mapped using EDS within a Carl Zeiss Ultra Plus Field Emission SEM, also at the NHM.

### Phylogenetic sequencing and analyses

Total genomic DNA was extracted from 64 *Sclerolinum* worm fragments: 27 Antarctic *Sclerolinum* sp., 15 *S. contortum* from the GoM, 21 *S. contortum* from Loki’s Castle, and one *S. contortum* individual from HMMV. Worm fragments with tentacles and forepart, and long worm fragments were selected for extractions to increase the likelihood of sampling from different individuals. DNA extractions of Antarctic and GoM specimens were performed using a Hamilton Microlab STAR Robotic Workstation combined with a DNeasy kit (Qiagen, Valencia, CA). Approximately 400 bp of the mitochondrial gene 16S, 600 bp of the mitochondrial gene COI, and 840–1370 bp of the nuclear 18S gene were amplified (all primers used for PCRs and sequencing are listed in Additional file 4: Table S3). PCR mixtures for Antarctic and GoM specimens contained 1 μl of each primer (10 μM), 2 μl of DNA template, and 21 μl of *Taq* PCR Master Mix (Qiagen). The PCR profile was as follows: 94 °C/300 s, (94 °C/60s, 50 °C/60s, 72 °C/120 s)*35 cycles, 72 °C/300 s. PCR products were visualised on 1.5 % agarose gels following electrophoresis, and sequenced using an Applied Biosystems 3730XL DNA Analyser at the NHM. DNA extraction and PCR of Arctic specimens (Loki’s Castle and HMMV) were carried out at the Biodiversity Laboratories, University of Bergen (BDL, DNA-lab section, Department of Biology) where an Applied Biosystems 3730XL DNA Analyser was used for sequencing. The PCR mixtures for amplification of 16S and COI contained 1 μl of each primer (10 μM), 1 μl of DNA template, 2.5 μl Qiagen CoralLoad buffer (10x), 1 μl Qiagen MgCl (25 μM), 2 μl dNTPs (TaKaRa; 2.5 μM of each dNTP), 0.15 μl TaKaRa HS taq, and 16.35 μl PCR water. The PCR profile for 16S was as follows: 95 °C/300 s, (95 °C/30s, 50 °C/30s, 72 °C/90s)*35 cycles, 72 °C/600 s, while the following profile was used for COI: 95 °C/300 s, (95 °C/45 s, 45 °C/45 s, 72 °C/60s)*5 cycles, (95 °C/45 s, 51 °C/45 s, 72 °C/60s)*35 cycles, 72 °C/600 s. In total, 16S was sequenced for 28 worm fragments, COI for 64, and 18S for two worm fragments.

Molecular phylogenetic analyses were performed using a combined dataset of 16S, COI and 18S sequences for members of the family Siboglinidae. A total of 44 terminal taxa were included in the analyses, of which five were *Sclerolinum*, and 39 were from other Siboglinidae genera. For the above analyses 111 sequences were obtained from NCBI Genbank, accession numbers for which are listed in Additional file 5: Table S4. The sabellid *Sabella pavonina* and spionid *Malacoceros fuliginosus* were used as outgroup taxa, of which *M. fuliginosus* was used to root the tree. Outgroup choice was based on the analyses of Rousset et al. [81] and Weigert et al. [82]. Overlapping sequence fragments were concatenated into consensus sequences using Geneious [83], and aligned using the following programs (provided as plug-ins in Geneious): MUSCLE for COI [84], and MAFFT for 18S and 16S [85]. The evolutionary models used for each gene were selected using jModelTest [86]. Based on the Akaike Information Criterion (AIC), the best fitting models of nucleotide substitution were TIM1 + I + G for COI and 18S, and TIM2 + G for 16S. As the model GTR + I + G is the closest approximation of the TIM models available in MrBayes, the GTR + I + G model was used for all three genes in the combined analysis. A Bayesian molecular phylogenetic analysis was conducted using MrBayes 3.1.2 [87]. Analyses of the combined dataset were run three times for 10,000,000 generations, with 2,500,000 generations discarded as burn-in. Genetic distances for the COI gene within the genus *Sclerolinum* were calculated using the K2P model, and *p*-distances were determined in MEGA 5.1 [88].

### Genetic diversity

A close relationship between Antarctic *Sclerolinum* sp. and *S. contortum* was detected from the above investigations, therefore an additional alignment was used for a phylogenetic analysis using a total of 68 COI *Sclerolinum* sp. sequences (*Sclerolinum brattstromi*, Kushiro-SK-2003 *Sclerolinum* sp., Loihi Seamount *Sclerolinum* sp., 27 Antarctic *Sclerolinum* sp., 15 GoM, 21 Loki’s Castle, and 2 HMMV *S. contortum*) for which two additional siboglinid COI sequences (*Riftia pachyptila* and *Siboglinum ekmani*) were used as outgroups. The alignment was trimmed to standardise sequence lengths, and the analysis was performed in the same way as the combined analysis outlined above. In addition, a haplotype distribution was created using only Antarctic *Sclerolinum* sp. and *S. contortum* sequences in TCS 1.21 [89] and drawn in PopART (http://popart.otago.ac.nz). Gaps were treated as missing data, and the connection limit was set to 95 %. There appeared to be little genetic differentiation between HMMV and Loki’s Castle *S. contortum* therefore sequences from these localities were subsequently pooled into one Arctic population, while GoM *S. contortum* and Antarctic *Sclerolinum* sp. were treated as two additional populations. Haplotype diversity, nucleotide diversity, and number of polymorphic sites were calculated within each population (Arctic, GoM and Antarctic) using DnaSP 5.10.1 [90]. Average genetic distances (K2P) within each population were calculated using MEGA 5.1 [88]. Pairwise *F*_*ST*_ values and an AMOVA were computed using Arlequin 3.5.1.3 [91]. The AMOVA was performed using K2P distances and 1000 permutations.

### Ethics statement

Ethics approval is not required for the collection and investigation of the morphology and DNA of annelid worms. Antarctic specimens were collected under the Foreign and Commonwealth Office Antarctic permit number S5-4/2010 issued to National Marine Facilities for the JC55 research expedition. Permits were not required by the collectors of Arctic and Gulf of Mexico material.

### Availability of supporting data

Morphological data supporting the results of this article are included within Additional file 1: Table S1. Occurrence data on specimens used in this study (in DarwinCore Archive format) and additional data sets (DNA sequence alignments) are available in the figshare repository (http://dx.doi.org/10.6084/m9.figshare.1613855). DNA sequences are available in GenBank (http://www.ncbi.nlm.nih.gov/genbank/), with NCBI accession numbers detailed in Additional file 5: Table S4 (a sequence for each detected *Sclerolinum contortum* COI haplotype is available).

## Additional files

Additional file 1: Table S1. Comparison of morphological characters between Antarctic *Sclerolinum* and three populations of *S. contortum*. (DOCX 100 kb) Additional file 2: Table S2. P-distance (above diagonal) and K2P (below diagonal) genetic distances (in %) among the genus, as well as putative, *Sclerolinum*. (DOCX 49 kb) Additional file 3:
**Methods supplement.** Details on the collection of additional *Sclerolinum contortum* specimens for comparison with Antarctic material. (DOCX 73 kb) Additional file 4: Table S3. Primers used for PCR and sequencing. (DOCX 118 kb) Additional file 5: Table S4. Taxa used in Bayesian molecular analyses and GenBank accession numbers. (DOCX 118 kb)

## Abbreviations

16S: Mitochondrial large subunit ribosomal RNA
18S: Nuclear small subunit subunit ribosomal RNA
AIC: Akaike information criterion
AMOVA: Analysis of molecular variance
COI: Cytochrome oxidase subunit I
EDS: Energy dispersive x-ray spectroscopy
EPR: East Pacific Rise
GoM: Gulf of Mexico
HMMV: Håkon Mosby Mud Volcano
K2P: Kimura 2 Parameter
MAFFT: Multiple Alignment using Fast Fourier Transform
MUSCLE: Multiple Sequence Comparison by Log-Expectation
NHM, NHMUK: Natural History Museum, United Kingdom
SHRIMP: Seafloor High Resolution Imaging Platform
