# Supplementary material for: A chemosynthetic weed: the tubeworm Sclerolinum contortum is a bipolar, cosmopolitan species
Source: BMC Evol Biol. 2015 Dec 14;15:280. doi: 10.1186/s12862-015-0559-y (PMC4678467; doi:10.1186/s12862-015-0559-y)
Supplement: Additional file 1: Table S1. — Comparison of morphological characters between Antarctic Sclerolinum and three populations of S. contortum. (DOCX 100 kb) [file 12862_2015_559_MOESM1_ESM.docx]

**Additional file 1: Table S1.** Comparison of morphological characters between Antarctic *Sclerolinum* and three populations of *S. contortum*.

|  | *S. contortum* HMMV* | *S. contortum* Loki’s Castle | *S. contortum* GoM** | *Sclerolinum* sp. Antarctic |
| --- | --- | --- | --- | --- |
|  | n=18 | n=10 | n=5 | n=10 |
| Tube, general character | a - contorted,  p - straight | a- wavy to contorted,  p - straight | a - contorted,  p - straight | a- wavy,  p - straight |
| Tube diameter (mm) | 0.20-0.39 | 0.33-0.42 | 0.35-0.61 | 0.22-0.30 |
| Thickness of tube wall (µm) | 56 | 43 (mean) | - | 9 (mean) |
| Anterior zone breadth (mm) | 0.13-0.24 | 0.22-0.29 | - | 0.15-0.23 |
| Distance from apex of cephalic lobe to frenulum (mm) | 0.27–0.30 | 0.25-0.52 | 0.32–0.59 | 0.13-0.37 |
| Arrangement of frenular plaques | Dense row | Dense row but plaques occasionally missing | Dense row/  scattered | Dense row but plaques occasionally missing |
| Frenulum, position | d-l-(v) | d-l-(v) | d-l-v | d-l-(v) |
| Number of frenular plaques | 10–14 | 18-23 | 12–20 | 9-19 |
| Frenular plaques, shape | Oval | Roundish to elongated | Roundish to oval | Roundish to elongated |
| Frenular plaques, diameter (μm) | 22–41 | 18-72 | 21–85 | 14-46 |
| Dorsal furrow | Deep, narrow | Deep, wide or narrow | Deep, narrow | Deep, wide |
| Plaques of trunk, diameter (μm) | 29-41 | 20-61 | 30-50 | 12-43 |
| Transition between forepart and trunk | Abrupt | Abrupt | Abrupt | Abrupt |
| Forepart length (mm) | 2.3-4.8 | 5.2-9.0 | 3.5-6.4 | 1.7-4.8 |
| Trunk length (mm) | 30-50 | > 60.1 | 47.9-80.6 | > 47.2 |
| Opisthosoma length (mm) | 0.45-0.6 | - | 1.4-1.8 | - |
| Opisthosoma number of segments | 3-5 | - | 13-16 | - |
| Uncini of opisthosoma, diameter (μm) | 5.5-6.3 | - | 4.5-6.5 | - |
| Habitat | Mud sediments, mud volcano | Sediments close to hydrothermal vents | Mud sediments, cold seeps | Sediments weakly exposed to hydrothermal flow |

*(v) - middle ventral plaque often missing, a - anterior, p - posterior. *Data from* [1]*. **Data from* [2]*.*

**References**

1. Smirnov R V.: **Two new species of Pogonophora from the arctic mud volcano off northwestern Norway**. *Sarsia* 2000, **85**:141–150.

2. Eichinger I, Hourdez S, Bright M: **Morphology, microanatomy and sequence data of Sclerolinum contortum (Siboglindae, Annelida) of the Gulf of Mexico**. *Org Divers Evol* 2013, **13**:311–329.
