# Supplementary material for: A chemosynthetic weed: the tubeworm Sclerolinum contortum is a bipolar, cosmopolitan species
Source: BMC Evol Biol. 2015 Dec 14;15:280. doi: 10.1186/s12862-015-0559-y (PMC4678467; doi:10.1186/s12862-015-0559-y)
Supplement: Additional file 2: Table S2. — P-distance (above diagonal) and K2P (below diagonal) genetic distances (in %) among the genus, as well as putative, Sclerolinum. (DOCX 49 kb) [file 12862_2015_559_MOESM2_ESM.docx]

**Additional file 2: Table S2.** P-distance (above diagonal) and K2P (below diagonal) genetic distances (in %) among the genus, as well as putative, *Sclerolinum*.

|  | Kushiro SK2003 | Loihi Seamount | *S. brattstromi* | *S. contortum* HMMV | *S. contortum* Loki’s Castle | *S. contortum* GoM | *Sclerolinum* sp. Antarctic |
| --- | --- | --- | --- | --- | --- | --- | --- |
| Kushiro SK2003 |  | 18.5 | 15.2 | 14.9 | 14.9 | 15.4 | 15.7 |
| Loihi Seamount | 21.6 |  | 15.7 | 15.2 | 15.2 | 15.2 | 14.9 |
| *S. brattstromi* | 17.1 | 17.8 |  | 9.4 | 9.4 | 8.8 | 9.4 |
| *S. contortum* HMMV | 16.7 | 17.0 | 10.2 |  | 0.0 | 0.8 | 1.4 |
| *S. contortum* Loki’s Castle | 16.7 | 17.0 | 10.2 | 0.0 |  | 0.8 | 1.4 |
| *S. contortum* GoM | 17.5 | 17.0 | 9.5 | 0.8 | 0.8 |  | 0.8 |
| *Sclerolinum* sp. Antarctic | 17.8 | 16.7 | 10.2 | 1.4 | 1.4 | 0.8 |  |
