# Supplementary material for: A chemosynthetic weed: the tubeworm Sclerolinum contortum is a bipolar, cosmopolitan species
Source: BMC Evol Biol. 2015 Dec 14;15:280. doi: 10.1186/s12862-015-0559-y (PMC4678467; doi:10.1186/s12862-015-0559-y)
Supplement: Additional file 3: — Methods supplement. Details on the collection of additional Sclerolinum contortum specimens for comparison with Antarctic material. (DOCX 73 kb) [file 12862_2015_559_MOESM3_ESM.docx]

**Additional file 3: Methods supplement.** Details on the collection of additional *Sclerolinum contortum* specimens for comparison with Antarctic material.

Loki’s Castle specimens (donated by Hans Tore Rapp) were collected during R/V G. O. Sars cruises during 2008-9 (samples S1-S13 were collected on ROV dive 8 [07/07/2009], 73°33.97’N 08°09.51’E, depth 2357 m; samples S17-24, and 30-31 were collected on ROV dive 11 [14/07/2008], 73°33.97'N-73°34.10'N 8°09.51'E-8°09.38'E, depth unknown). A single Håkon Mosby Mud Volcano (HMMV) specimen was used for DNA studies (S28) and was collected during 2010 on the R/V G. O. Sars (ROV dive 2 [10/07/2010], 71°59.852'N-71°59.993'N 14°43.975'E-14°43.897'E, depth 1262). Additional dried empty *S. contortum* tubes from HMMV (donated by Ann Andersen) were also used for comparisons with *Sclerolinum* tubes from other locations. These were collected during the June 2006 VICKING cruise on board R/V *Pourquoi pas?* (sample no. VI-103B, KGS 5, 72° 00.0785N, 14°43.3477E, 1270 m depth). Gulf of Mexico specimens (donated by Monika Bright) consisting of tubes with and without tissue and preserved in ethanol, were collected during NOAA *Ronald H. Brown* expedition no. RB-07-04 during June 2007 (Jason Dive 275, WR269, 26°41N, 91°39W, 1954 m depth).
