# Supplementary material for: A chemosynthetic weed: the tubeworm Sclerolinum contortum is a bipolar, cosmopolitan species
Source: BMC Evol Biol. 2015 Dec 14;15:280. doi: 10.1186/s12862-015-0559-y (PMC4678467; doi:10.1186/s12862-015-0559-y)
Supplement: Additional file 4: Table S3. — Primers used for PCR and sequencing. (DOCX 118 kb) [file 12862_2015_559_MOESM4_ESM.docx]

**Additional file 4: Table S3**. Primers used for PCR and sequencing.

| Primer | Sequence 5’-3’ | References |
| --- | --- | --- |
| 16 AnnF | GCGGTATCCTGACCGTRCWAAGGTA | [1] |
| 16 AnnR | TCCTAAGCCAACATCGAGGTGCCAA | [1] |
| 16Sar | CGCCTGTTTATCAAAAACAT | [2] |
| 16Sbr | CTCCGGTTTGAACTCAGATC | [2] |
| PolyLCO | GAYTATWTTCAACAAATCATAAAGATATTGG | [3] |
| PolyHCO  LCO1490  HCO2198 | TAMACTTCWGGGTGACCAAARAATCA  GGTCAACAAATCATAAAGATATTGG  TAAACTTCAGGGTGACCAAAAAATCA | [3]  [4]  [4] |
| 18SA | AYCTGGTTGATCCTGCCAGT | [5] |
| 18SB | ACCTTGTTACGACTTTTACTTCCTC | [6] |
| 620F | TAAAGYTGYTGCAGTTAAA | [6] |
| 1324R | CGGCCATGCACCACC | [7] |

**References**

1. Sjölin E, Erséus C, Källersjö M: **Phylogeny of Tubificidae (Annelida, Clitellata) based on mitochondrial and nuclear sequence data.** *Mol Phylogenet Evol* 2005, **35**:431–41.

2. Palumbi SR: **Nucleic acid II: the polymerase chain reaction**. In *Molecular Systematics*. Edited by Hillis DM, Moritz C, Mable BK. Sunderland, MA: Sinauer Associates; 1996:205–247.

3. Carr CM, Hardy SM, Brown TM, Macdonald TA, Hebert PDN: **A tri-oceanic perspective: DNA barcoding reveals geographic structure and cryptic diversity in Canadian polychaetes**. *PLoS One* 2011, **6**:e22232.

4. Folmer O, Black M, Hoeh W, Lutz R, Vrijenhoek R: **DNA primers for amplification of mitochondrial cytochrome c oxidase subunit I from diverse metazoan invertebrates**. *Mol Mar Biol Biotechnol* 1994, **3**:294–299.

5. Medlin L, Elwood HJ, Stickel S, Sogin ML: **The characterization of enzymatically amplified eukaryotic 16S-like rRNA-coding regions**. *Gene* 1988, **71**:491–499.

6. Nygren A, Sundberg P: **Phylogeny and evolution of reproductive modes in Autolytinae (Syllidae, Annelida)**. *Mol Phylogenet Evol* 2003, **29**:235–249.

7. Cohen BL, Gawthrop A, Cavalier-Smith T: **Molecular phylogeny of brachiopods and phoronids based on nuclear-encoded small subunit ribosomal RNA gene sequences**. *Philos Trans R Soc B Biol Sci* 1998, **353**:2039–2061.
