# Supplementary material for: A chemosynthetic weed: the tubeworm Sclerolinum contortum is a bipolar, cosmopolitan species
Source: BMC Evol Biol. 2015 Dec 14;15:280. doi: 10.1186/s12862-015-0559-y (PMC4678467; doi:10.1186/s12862-015-0559-y)
Supplement: Additional file 5: Table S4. — Taxa used in Bayesian molecular analyses and GenBank accession numbers. (DOCX 118 kb) [file 12862_2015_559_MOESM5_ESM.docx]

**Additional file 5: Table S4**. Taxa used in Bayesian molecular analyses and GenBank accession numbers.

| **Terminal taxa** | **COI** | **18S** | **16S** |
| --- | --- | --- | --- |
| *Malacoceros fuliginosus* | EF432015 | EF446961 | EF431961 |
| *Sabella pavonina* | KF369181 | U67144 | AY340482 |
| *Alaysia* sp. | FJ667536 | FM995545 | --- |
| *Escarpia laminata* | KC357324 | --- | HE974467 |
| *Escarpia southwardae* | KC870958 | --- | KC870952 |
| *Escarpia spicata* | KC870961 | AF168741 | KC870943 |
| *Galathealinum brachiosum* | U74066 | AF168738 | AF315040 |
| Kushiro-SK-2003 pogonophoran | D50598 |  |  |
| *Lamellibrachia anaximandri* | KC832757 | --- | KF199248 |
| *Lamellibrachia barhami* | AY129145 | --- | AF315047 |
| *Lamellibrachia columna* | DQ996645 | FJ347679 | FJ347646 |
| *Lamellibrachia luymesi* | GU059233 | --- | GU068251 |
| ‘Loihi Seamount’ pogonophoran | PSU74068 |  |  |
| *Oasisia alvinae* | AY646020 | AF168743 | AF315052 |
| *Oligobrachia haakonmosbiensis* | FM178482 | AM883186 | --- |
| *Osedax antarcticus* | KF444427 | KF444420 | KF444418 |
| *Osedax crouchi* | KJ598038 | KJ598035 | KJ598032 |
| *Osedax deceptionensis* | KF444428 | KF444421 | KF444419 |
| *Osedax frankpressi* | FJ347607 | AY577885 | AY577876 |
| *Osedax japonicus* | FM998111 | FM995535 | --- |
| *Osedax mucofloris* | HM045513 | AY941263 | --- |
| *Osedax nordenskjoeldi* | KJ598039 | KJ598036 | KJ598033 |
| *Osedax rogersi* | KJ598040 | KJ598037 | KJ598034 |
| *Osedax roseus* | JF509949 | FJ347683 | FJ347657 |
| *Osedax rubiplumus* | EU852488 | AY577894 | AY577878 |
| *Osedax* sp.'greenpalp' | FJ347641 | FJ347694 | FJ347655 |
| *Osedax* sp. 'nudepalpA' | FJ347623 | FJ347687 | FJ347653 |
| *Osedax* sp. 'nudepalpC' | EU267676 | FJ347688 | FJ347650 |
| *Osedax* sp. 'orangecollar' | FJ431203 | FJ347690 | FJ347661 |
| *Osedax* sp. 'sagami3' | FM998080 | FM995537 | --- |
| *Osedax* sp. 'spiral' | FJ347637 | FJ347693 | FJ347647 |
| *Osedax* sp. 'whitecollar' | FJ347613 | FJ347684 | FJ347659 |
| *Osedax* sp. 'yellowcollar' | EU223338 | FJ347689 | FJ347660 |
| *Osedax* sp. 'yellowpatch' | FJ347618 | FJ347685 | FJ347654 |
| *Paraescarpia echinospica* | D50594 | FM995546 | --- |
| *Ridgeia piscesae* | EU190708 | AF168744 | AF315054 |
| *Riftia pachyptila* | FJ667529 | AF168745 | AF315050 |
| *Sclerolinum brattstromi* | FJ347644 | FJ347680 | FJ347645 |
| *Sclerolinum contortum* GoM | *KU214839 | --- | JX013982 |
| *Sclerolinum contortum* HMMV | FM178480 | AM883187 | JX013984 |
| *Sclerolinum contortum* Loki’s Castle | *KU214833 | --- | *KU214831 |
| *Sclerolinum contortum* Antarctica | *KU214832 | *KU214829 | *KU214830 |
| *Siboglinum ekmani* | KF444429 | AF315062 | AF315038 |
| *Siboglinum fiordicum* | --- | AF315060 | AF315039 |
| *Spirobrachia* sp. | FJ480372 | AF168740 | AF315036 |
| *Tevnia jerichonana* | FJ667531 | AF168746 | --- |

Notes: --- no sequence available, asterisk (*) denotes sequences provided as part of this study, grey highlight denotes sequences used for *Sclerolinum* COI only Bayesian analysis **(Figure 8)**.

**COI sequences submitted to GenBank (for each *Sclerolinum contortum* haplotype) and accession numbers:**

Sclerolinum contortum G44 Antarctica-haplotype 1 (in table above)

Sclerolinum contortum S1 Loki’s Castle-haplotype 1 (in table above)

Sclerolinum contortum S12 Loki’s Castle-haplotype 2 KU214834

Sclerolinum contortum S20 Loki’s Castle-haplotype 3 KU214835

Sclerolinum contortum S23 Loki’s Castle-haplotype 4 KU214836

Sclerolinum contortum S24 Loki’s Castle-haplotype 5 KU214837

Sclerolinum contortum S28 HMMV-haplotype 1 KU214838

Sclerolinum contortum G69 GoM-haplotype 1 KU214840

Sclerolinum contortum G72 GoM-haplotype 2 KU214841

Sclerolinum contortum G73 GoM-haplotype 3 KU214842

Sclerolinum contortum G74 GoM-haplotype 4 KU214843

Sclerolinum contortum G76 GoM-haplotype 5 KU214844

Sclerolinum contortum G78 GoM-haplotype 6 KU214845

Sclerolinum contortum G68 GoM-haplotype 7 (in table above)
